# Targeting of glioma stem-like cells with a parthenolide derivative ACT001 through inhibition of AEBP1/PI3K/AKT signaling

**DOI:** 10.7150/thno.49250

**Published:** 2021-01-01

**Authors:** Yanli Hou, Bowen Sun, Wenxue Liu, Bo Yu, Qiqi Shi, Fei Luo, Yongrui Bai, Haizhong Feng

**Affiliations:** 1 Department of Radiotherapy, Ren Ji Hospital, School of Medicine, Shanghai Jiao Tong University, Shanghai 200127, China; 2 State Key Laboratory of Oncogenes and Related Genes, Renji-Med X Clinical Stem Cell Research Center, Ren Ji Hospital, Shanghai Cancer Institute, School of Medicine, Shanghai Jiao Tong University, Shanghai 200127, China

**Keywords:** ACT001, glioma stem-like cells, AEBP1, AKT, PI3K, SHP099

## Abstract

Glioblastoma (GBM) is the most lethal primary brain tumor in adults with a median survival of around 15 months. A potential treatment strategy involves targeting glioma stem-like cells (GSCs) that are able to initiate, maintain, and repopulate the tumor mass. Here, we identify ACT001, a parthenolide derivative, targeting GSCs through regulation of adipocyte enhancer binding protein 1 (AEBP1) signaling.

**Methods:** The effects of ACT001 on cell survival of normal human astrocytes (NHA) and patient-derived glioma stem-like cells (GSCs) were evaluated. RNA-Seq were performed to detect differentially expressed genes. ACT001 efficacy as a single agent or in combination with SHP-2 inhibitor SHP099 was assessed using a GSC orthotopic xenograft model.

**Results:** GSCs exhibit high response to ACT001 in compared with normal human astrocytes. AEBP1 is a putative target of ACT001 by RNA-Seq analysis, which expression associates with prognosis of GBM patients. Knockdown of AEBP1 inhibits GSC proliferation and glioma sphere formation. Treatment with ACT001 or PI3K inhibitor or AEBP1 depletion would impair AKT phosphorylation and GSC proliferation, whereas constitutive AKT activation rescues ACT001 treatment or AEBP1 depletion-inhibited cell proliferation. Moreover, ACT001 blocks TGF-β-activated AEBP1/AKT signaling in GSCs. ACT001 exhibits antitumor activity either as a single agent or in combination with SHP099, which provides significant survival benefits for GSC tumor xenograft-bearing animals.

**Conclusions:** Our data demonstrate AEBP1 as a new druggable target in GBM and ACT001 as a potential therapeutic option for improving the clinical treatment of GBM in combination with SHP099.

## Introduction

Glioblastoma (GBM) is the most common and aggressive primary brain cancer with poor prognosis 1, 2. Typically, GBM patients underwent surgical resection, combined with postoperative radiotherapy and chemotherapy 3. Chemotherapies including cisplatin and temozolomide (TMZ) are important clinical adjuvant therapy 4, 5. Given chemoresistance, GBM patients still have a poor median survival rate of 15 months. Glioma stem-like cells (GSCs) are recognized as a special population of GBM cells that contributes to tumorigenesis, radiochemoresistance, and recurrence 6-9. Developing new therapeutic drugs combined with conventional therapy is strongly needed for GBM patients.

ACT001 (also known as dimethylaminomicheliolide, DMAMCL) is developed from parthenolide, a sesquiterpene lactone by Accendatech Co., Ltd (Tianjin, China) 10. ACT001 showed anti-tumor functions in various cancers, including leukemia 11, 12 and breast cancer 13. ACT001 has been certified as an orphan drug by both the Food and Drug Administrations in the United States and in the European Union 10, 11. ACT001 was shown to selectively inhibit acute myelogenous leukemia stem and progenitor cells 11, 12. More important, ACT001 resulted in good control of GBM growth in China and Australia in Phase I clinical trials 12, and now it is currently undergoing Phase II clinical trials. ACT001 showed synergistic effects with combination of cisplatin by inhibition of PI3K/AKT signaling pathway in GBM 14. However, the mechanisms by which ACT001 inhibits glioma progression is not clear, which limits the application of this new therapeutic target.

Adipocyte enhancer binding protein 1 (AEBP1) was at first discovered as a transcriptional repressor that binds to the AE-1 element of the ap-2 gene, coding for the fatty acid binding protein (FABP4) 15. Then, AEBP1 was identified as an oncogenic transcription factor 16-18. AEBP1 upregulation was observed in prostate cancer 16, which conferred to acquired resistance to BRAF (V600E) inhibition in melanoma. Through upregulation of sonic Hedgehog and NF-kappa B (NF-κB) pathway, AEBP1 functioned as a pro-inflammatory mediator and promoted mammary cell hyperplasia 17. In gliomas, AEBP1 up-regulated PI3KCB transcription, leading to enhanced AKT phosphorylation and cell proliferation 18.

Here, we investigated whether AEBP1 is a new target of ACT001 in gliomas with activating AKT signaling. We determined the response of AEBP1 knockdown on ACT001 response in GSCs. Treatment with ACT001 either as a single agent or in combination with SHP099 was further performed using an orthotopic xenograft model of GSC.

## Materials and Methods

### Cell culture

Normal human astrocytes from Lonza were maintained in Dulbecco's Modified Eagle's Medium supplemented with 10% fetal bovine serum (FBS) and 1% penicillin/streptomycin (GIBCO). Patient-derived glioma stem cell (GSC) lines, GSC 1123 and R39, were from Dr. Ichiro Nakano 19 or our collections 20. GSC cells were maintained in DMEM/F12 (GIBCO) supplemented with B27 (2%), heparin (5 mg/ml), basic FGF (20 ng/ml), EGF (20 ng/ml) and 1% penicillin/streptomycin.

### shRNA knockdown (KD) and infection

DNA and packaging plasmids were transduced into HEK293T cells using Lipofectamine 2000 reagent according to manufacturer's instruction (Invitrogen 52758). After 48-h transfection, the viral-containing supernatants were filtered and added into the culture media supplemented with 8 mg/ml polybrene. AEBP1 and AKT short hairpin RNAs (shRNAs) and control shRNAs were purchased from Shanghai GeneChem Company.

### Chemical

Parthenolide derivative (ACT001) was provided by Accendatech Co., Ltd (Tianjin, China) and dissolved in double-distilled (dd)H_2_O 10. SHP099 (HY-100388) were purchased from MedChemExpress and dissolved in ddH_2_O 21.

### Cell proliferation and viability assays

Cells were plated in triplicate wells of a 96-well microplate (6000 cells/well). Cell proliferation analysis was performed using a WST-1 assay kit (BioVision Inc.). After 24 h, cells were treated with vehicle (ddH_2_O) or ACT001 from 0.1 to 100 μM for 72 h and cell viability was then analyzed using the WST-1 assay kit. Half-maximal inhibitory concentration (IC_50_) values were calculated from fitted concentration-response curves obtained from at least 3 independent experiments. IC_50_ values were determined using GraphPad Prism 7 nonlinear regression curve fit.

### Colony formation and limiting dilution glioma sphere-forming assays

Colony formation and limiting dilution assays were performed as we previously described 8, 22. In brief, dissociated cells from glioma spheres were seeded in 96-well plates containing GSC cell culture medium (20-200 cells per well). After 7 days, each well was examined for formation of tumor spheres. Stem cell frequency using extreme limiting dilution analysis was calculated as described in http://bioinf.wehi.edu.au/software/elda/.

### Isobologram and combination index analysis

Cells were seeded in 96-well plates and treated with ACT001, SHP009 or combination for 72 h. Media were replaced with fresh media containing increasing concentrations of ACT001 (ranging from 0 μM to 64 μM) and SHP099 (ranging from 0 μM to 19.2 μM), with each concentration tested in triplicate wells. The concentrations used corresponded to 0.0625, 0.125, 0.25, 0.5, 1, 2, 4 times the IC50 of each agent. The concentration of the single drug that inhibits 50% of cell proliferation (IC50) was determined by fitting the dose-response curve utilizing the CompuSyn software. To calculate the Combination Index (CI), using the method of constant ratio drug combination proposed by Chou and Talalay 23, 24 and described in result. The CI was calculated using the formula: C.I. = C_A_, _X_/IC_X_, _A_+C_B_, _X_/IC_X_, _B_, where C_A_, _X_ and C_B_, _X_ are the concentration of ACT001 and SHP009 used in combination to achieve 50% drug effect. IC_X_, _A_ and IC_X_, _B_ are the concentrations for single agents to achieve the same effect. A CI of less then, equal to, or more than 1 indicates synergic, additive or antagonistic effect, respectively.

### Cell cycle analysis

After ACT001 treatment, culture supernatant and trypsinized cells were combined and centrifuged. After two washes in cold PBS, cells were fixed with 70% ethanol overnight at 4ºC and resuspended in 0.5 ml staining solution supplemented with 10 μl propidium iodine (PI) and 10 μl RNase A (YEASON). After incubation for 30 min at 37ºC in the dark, analysis was performed on a FACS ACCuri C6 (BD Biosciences).

### RNA Isolation, RNA Sequencing (RNA-Seq), and quantitative Real-time PCR (qRT-PCR)

Total RNA was extracted with TRIzol reagent (Invitrogen). RNA-Seq was performed as previously described 25 and the data had been deposited with the Gene Expression Omnibus under accession ID GSE157779. A Maxima First Strand cDNA Synthesis Kit (Thermo Fisher Scientific) was used and qRT-PCR was performed with Maxima SYBR Green qPCR Master Mix (Applied Biosystems) on a QuantStudio 6 Flex Real-Time PCR System (Applied Biosystems). The primers were listed: GAPDH, 5'-CATCACTGCC ACCCAGAAGACTG-3' and 5'-ATGCCAGTGAGCTTCCCGTTCAG-3'; AEBP1, 5'-GAGGAGTTGGAGGAGGAGTGGAC-3' and 5'-AGGAGGCTCGGATCTGGTTGTC-3'. GAPDH was used as the internal control.

### Western blotting (WB) analysis

Cells were lysed in a buffer (20 mM Tris-HCl, pH 7.5, 150 mM NaCl, 1 mM EDTA, 2 mM Na_3_VO_4_, 5 mM NaF, 1% Triton X-100 and protease inhibitor cocktail) at 4°C for 30 min. The protein concentrations of lysis were determined. Equal amounts of cell lysates were loaded. Western blotting (WB) were performed as previously described 25. Antibodies were used: Actin (Proteintech, 66009-1-lg), AEBP1 (Santa Cruz Biotechnology, 271374), phospho-AKT (Ser473) (Cell Signaling Technology, 4060), AKT (CST, 9272). The original WB images are shown in Supplementary Figure 2.

### Tumorigenicity Studies

All animal experiments were conducted in accordance with the animal use guidelines by the Shanghai Jiao Tong University Institutional Animal Care and Use Committee. Athymic nu/nu female mice aged 6 weeks (SLAC, Shanghai, P.R.C) were used. In total 2,000 GSCs were stereotactically implanted into the brain of the animals as previously described 20. A scheme of treatment was described in the corresponding figure legend. Mice were euthanized when neuropathological symptoms developed.

### Histology and immunohistochemistry (IHC)

IHC staining was performed as described previously 26. Optimal cutting temperature (OCT)-embedded sister sections of bearing glioma xenograft tumor mouse brains were separately stained with hematoxylin. IHC staining was performed with antibodies against AEBP1 (1:10), phosphor-AKT (1:100), Ki-67 (Thermo, MA5-14520, 1: 1000), and Nestin (Millipore, ABD69, 1: 100) as previously described 20.

### Statistical Analysis

All statistical analyses were performed using GraphPad Prism 7 software (GraphPad Software Inc.). A minimum of 3 independent biological replicates were analyzed using Student's two-tailed* t*-test or one-way ANOVA (Newman-Keuls post hoc test) as specified in the figure legends. Data are expressed as the mean ± SEM of 3 independent experiments. Survival analysis was performed using Kaplan-Meier analysis and the log-rank test. *p* < 0.05 was considered significant.

## Results

### GSCs are more responsive to ACT001 treatment

**Figure 1:**
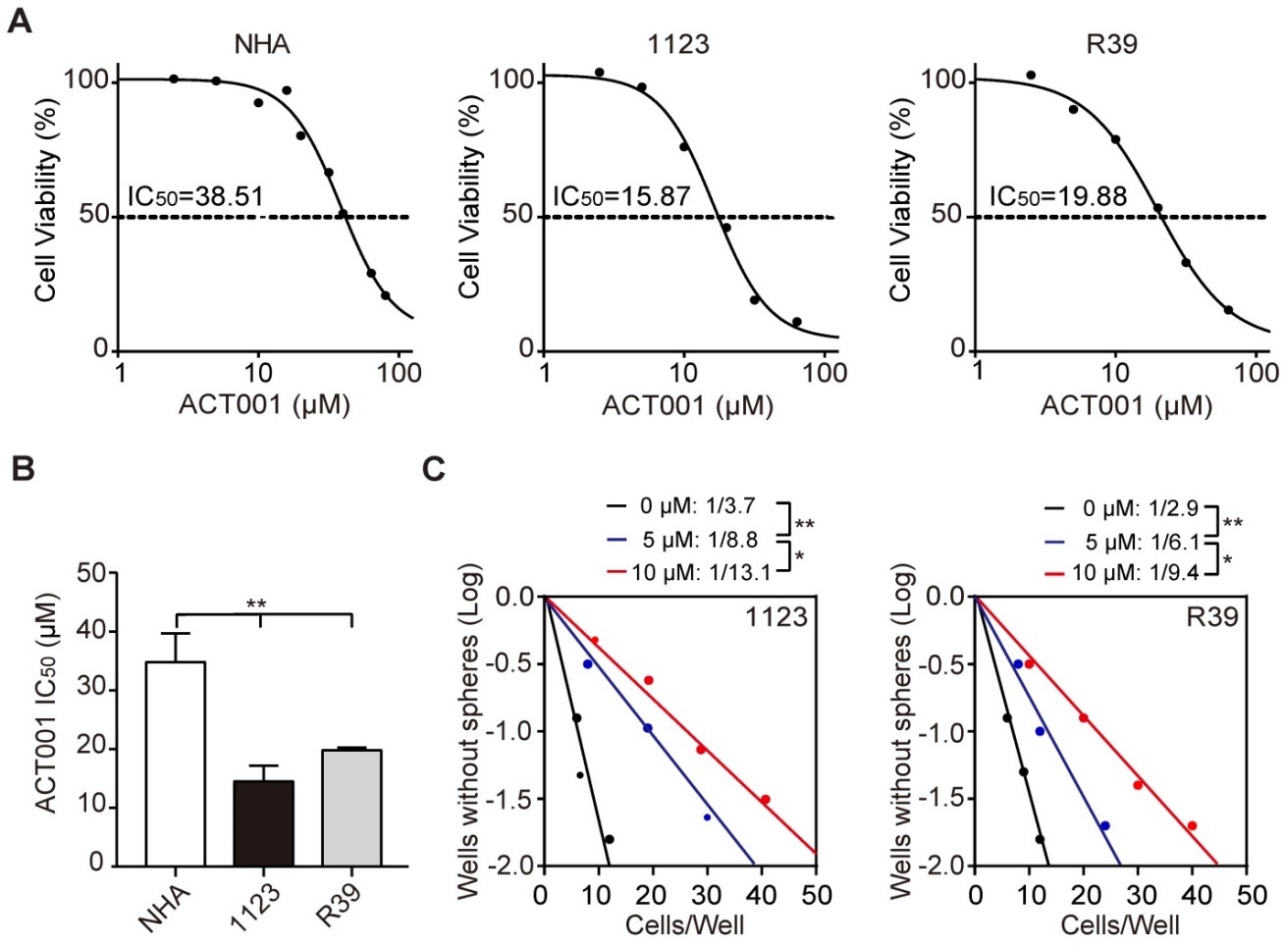
** GSCs are more responsive to ACT001 treatment than NHA. (A)** Viability of NHA, GSC 1123, and R39 cells at 72 h after treatment with ACT001 from 0.1 to 100 μM. **(B)** Comparison of ACT001 IC_50_ values in (**A**). **(C)** Effects of ACT001 in limiting dilution glioma sphere-forming assay of GSC 1123 or R39 cells. Data represent the mean ± SEM of replicates from 3 independent experiments. *p < 0.05; **p < 0.01, by one-way ANOVA or two-tailed *t*-test.

ACT001 is an orally available chemotherapeutic agent with IC_50_ of 27 μM and 21 μM against C6 and U87 glioma cells, respectively 10. To assess the effects of ACT001 on glioma stem-like cells (GSCs), we performed cell viability analysis and found that compared to normal human astrocytes (NHA), patient-derived GSC 1123 and R39 cells were more responsive to ACT001 (Figure 1A and 1B). ACT001 markedly inhibited glioma sphere formation in GSC 1123 and R39 cells (Figure 1C). We also performed flow cytometric analyses of cell cycle and found that compared the vehicle, ACT001 did not impair the cell cycle in NHA, GSC 1123, or R39 cells (Supplementary Figure 1). This data indicate that ACT001 is more selective for GSCs than NHA.

### ACT001 inhibits the expression of AEBP1, a prognostic factor of gliomas

**Figure 2:**
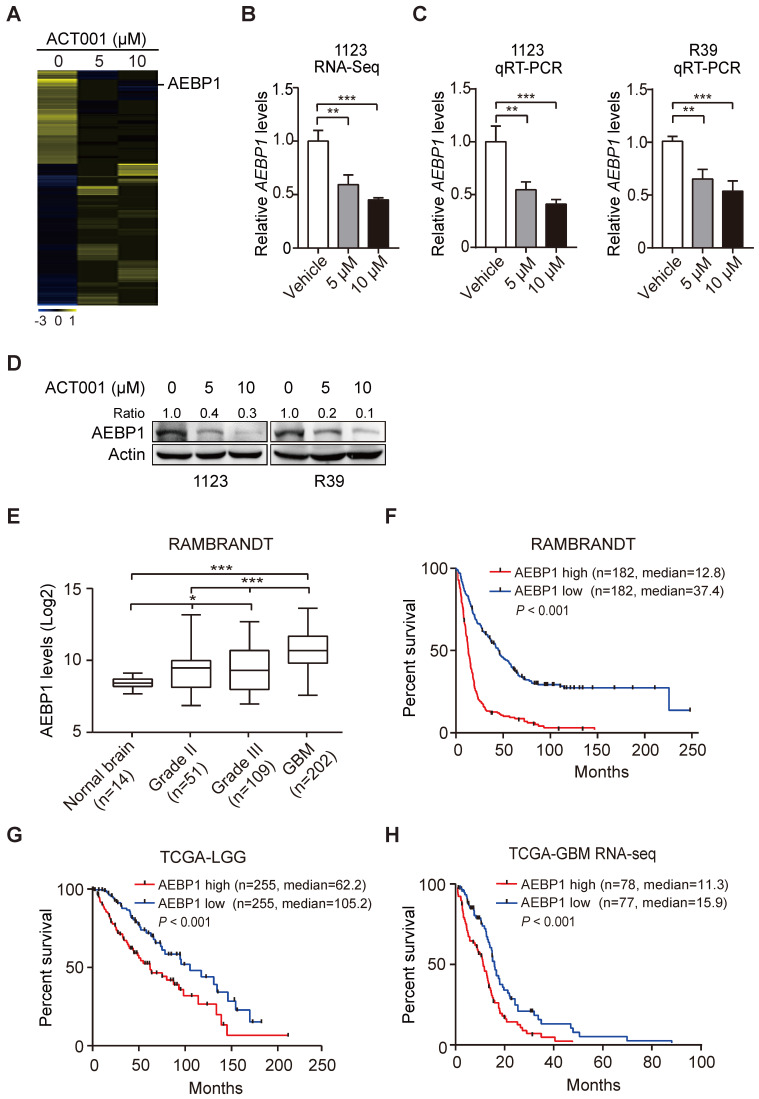
** ACT001 inhibits the expression of AEBP1, a prognostic factor of gliomas. (A)** Heatmap of RNA-Seq analysis of differentially expressed genes (2-fold change and false discovery rate < 0.05) in GSC 1123 cells treated with 0, 5, or 10 μM ACT001. **(B** and** C)** Expression levels of *AEBP1* in (**A**) determined by RNA-Seq (**B**) or qRT-PCR (**C**) assays. **(D)** Western blotting (WB) of AEBP1 protein expression in (**A**). **(E)** Expression levels of *AEBP1* is correlated with glioma progression. Expression data of *AEBP1* were download from the REMBRANDT dataset. Median survival (in months): low, 44.7; high, 12.8. **(F)** Kaplan-Meier analysis of patients with high versus low AEBP1-expressing glioma tumors from the REMBRANDT dataset. Median survival (in months): low, 37.4; high, 12.8. Black bars, censored data. **(G** and** H)** Kaplan-Meier analysis of patients with high versus low AEBP1-expressing LGG (**G**) and GBM (**H**) tumors from the TCGA datasets. Median survival (in months) in (**G**): low, 105.2; high, 62.2; in (**H**): low, 15.9; high, 11.3. Black bars, censored data. In **F**, **G**, and **H**, statistical analysis was performed by log-rank test. Data represent the mean ± SEM of replicates from three independent experiments. *p < 0.05, **p < 0.01, ***p < 0.001, by two-tailed *t*-test or one-way ANOVA.

To investigate the underlying mechanism by which GSCs are more responsive to ACT001 treatment, we employed RNA-Seq analysis in GSC 1123 cells treated with or without ACT001. Gene expression profile analysis identified 858 genes whose expression was significantly reduced by ACT001 treatment and 1075 genes whose expression was markedly increased by ACT001 treatment (false discovery rate < 0.05 and a folder change > 2) (Figure 2A). *AEBP1* is a top one in ACT001 down-regulated genes (Figure 2A). Given that AEBP1 is important for various cancers 16, 17, including glioma 18, we further investigated if it is a target of ACT001 in GSCs. As shown in Figure 2B, the expression level of *AEBP1* was significantly decreased by both 5 and 10 μM ACT001 treatment. This result was further validated by qRT-PCR (Figure 2C) and Western blotting (WB) analyses (Figure 2D) in GSC 1123 and R39 cells. This data suggests that AEBP1 is a putative target of ACT001 in GSCs.

We then assessed the expression levels of AEBP1 in normal brain tissues and clinical specimens of glioma patients. We downloaded the RAMBRANDT dataset (http://gliovis.bioinfo.cnio.es/) and found that compared to normal brain tissues, the expression levels of *AEBP1* mRNA was significantly high in low grade tumors (Grade II and III) and GBM. Compared to low grade gliomas (Grade II and III), the expression levels of *AEBP1* mRNA was markedly upregulated in GBM. This data suggests that the expression of *AEBP1* is correlated with glioma progression.

Next, we determined the relationship of *AEBP1* expression and glioma patient survival using the RAMBRANDT and the Cancer Genome Atlas (TCGA) datasets. As shown in Figure 2F, Kaplan-Meier survival analysis revealed a statistically significant worse prognosis for glioma patients with high *AEBP1* (> median level) compared to those with low levels of *AEBP1* (< median level) in the RAMBRANDT dataset. The median patient survival times of these patients were 12.8 and 37.4 months, respectively. Kaplan-Meier survival analysis of the TCGA low-grade glioma (TCGA-LGG) and TCGA GBM RNA-Seq datasets further indicated that AEBP1 is a prognostic factor for glioma patients (Figures 2G and 2H). These data demonstrate that ACT001 may inhibit glioma progression through targeting AEBP1, a prognostic factor for gliomas.

### ACT001 targets AEBP1 to decrease AKT phosphorylation and GSC proliferation

**Figure 3:**
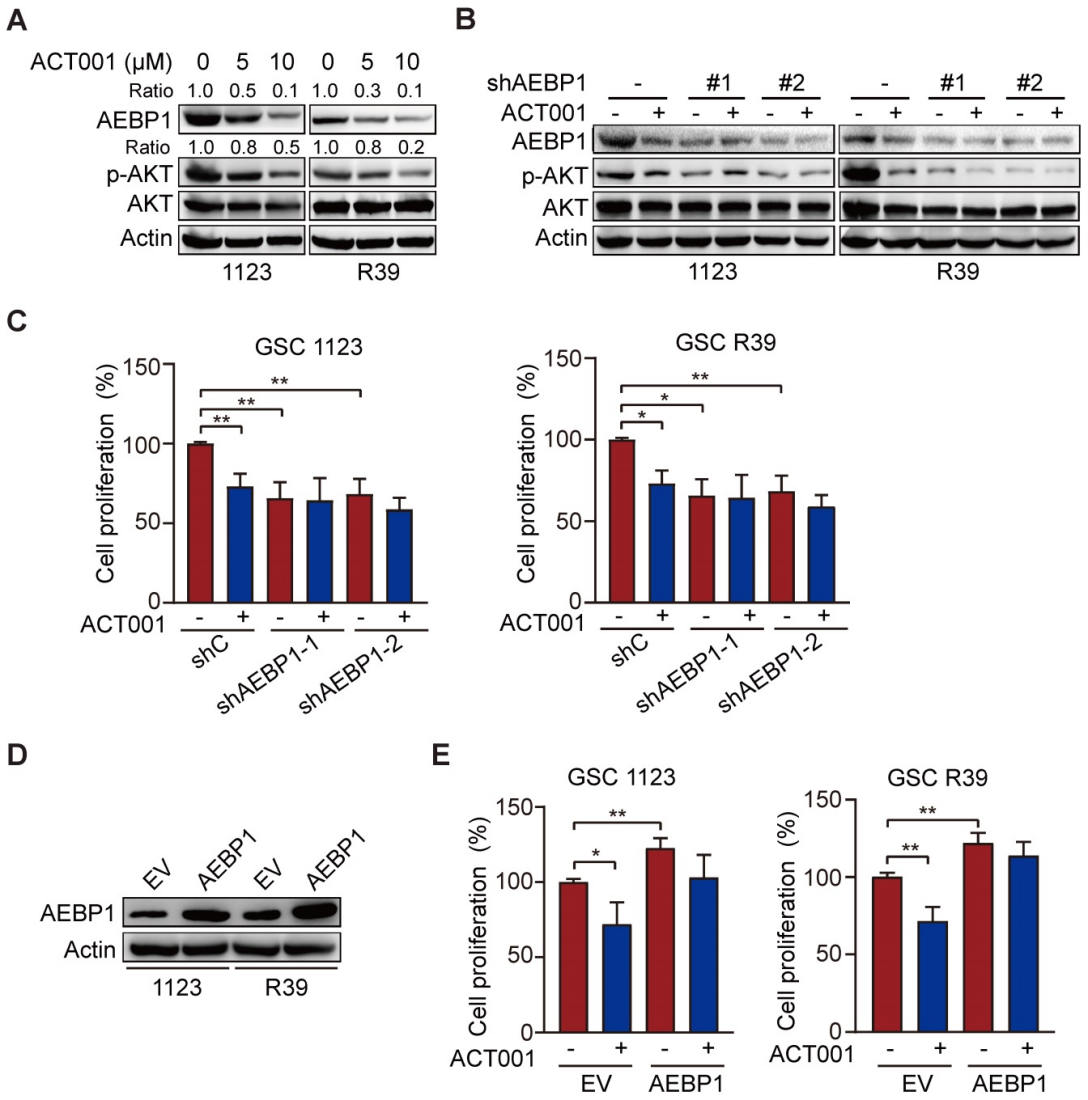
** ACT001 targets AEBP1 to decrease AKT phosphorylation and GSC proliferation. (A)** WB of AEBP1 expression and phosphorylation AKT (p-AKT). GSC 1123 and R39 cells were treated with 0, 5, or 10 μM ACT001 for 24 h. **(B)** Effect of *AEBP1* knockdown (KD) on ACT001-inhibited p-AKT. *AEBP1* was knocked down with two different shRNAs in GSC 1123 and R39 cells. **(C)** Effect of *AEBP1* knockdown (KD) on ACT001-inhibited GSC proliferation. **(D)** WB of AEBP1 overexpression in GSC 1123 and R39 cells. EV, an empty vector. **(E)** Effect of AEBP1 overexpression on GSC cell proliferation. Data represent the mean ± SEM of replicates from three independent experiments. *, *p* < 0.05, **p < 0.01, by one-way ANOVA.

ACT001 inhibits glioma cell proliferation by inhibiting PI3K/AKT activation 14 and AEBP1 activates PI3K/AKT signaling through upregulation of PI3KCB transcription 18. Thus, we investigated whether AEBP1 mediates ACT001 response through PI3K/AKT signaling. We first treated GSC 1123 and R39 cells with ACT001 and detected AEBP1 expression and AKT phosphorylation (p-AKT). As shown in Figure 3A, consistent with the above, ACT001 treatment decreased AEBP1 protein expression. ACT001 treatment also significantly attenuated p-AKT in GSC 1123 and R39 cells (Figure 3A). Then, AEBP1 was knocked down by two different shRNAs (Figure 3B). Compared with the shRNA control, *AEBP1* knockdown (KD) markedly decreased p-AKT (Figure 3B) and cell proliferation (Figure 3C) in GSC 1123 and R39 cells. However, *AEBP1* KD in combination with ACT001 treatment did not further reduced p-AKT and cell proliferation compared to ACT001 treatment (Figures 3B and 3C). Additionally, ectopic expression of AEBP1 increased cell proliferation and attenuated ACT001-inhibited cell proliferation in GSC 1123 and R39 cells (Figures 3D and 3E). These data suggest that ACT001 targets AEBP1 to decrease p-AKT and GSC proliferation.

### ACT001 inhibits GSC proliferation through AEBP1/PI3K/AKT signaling

**Figure 4:**
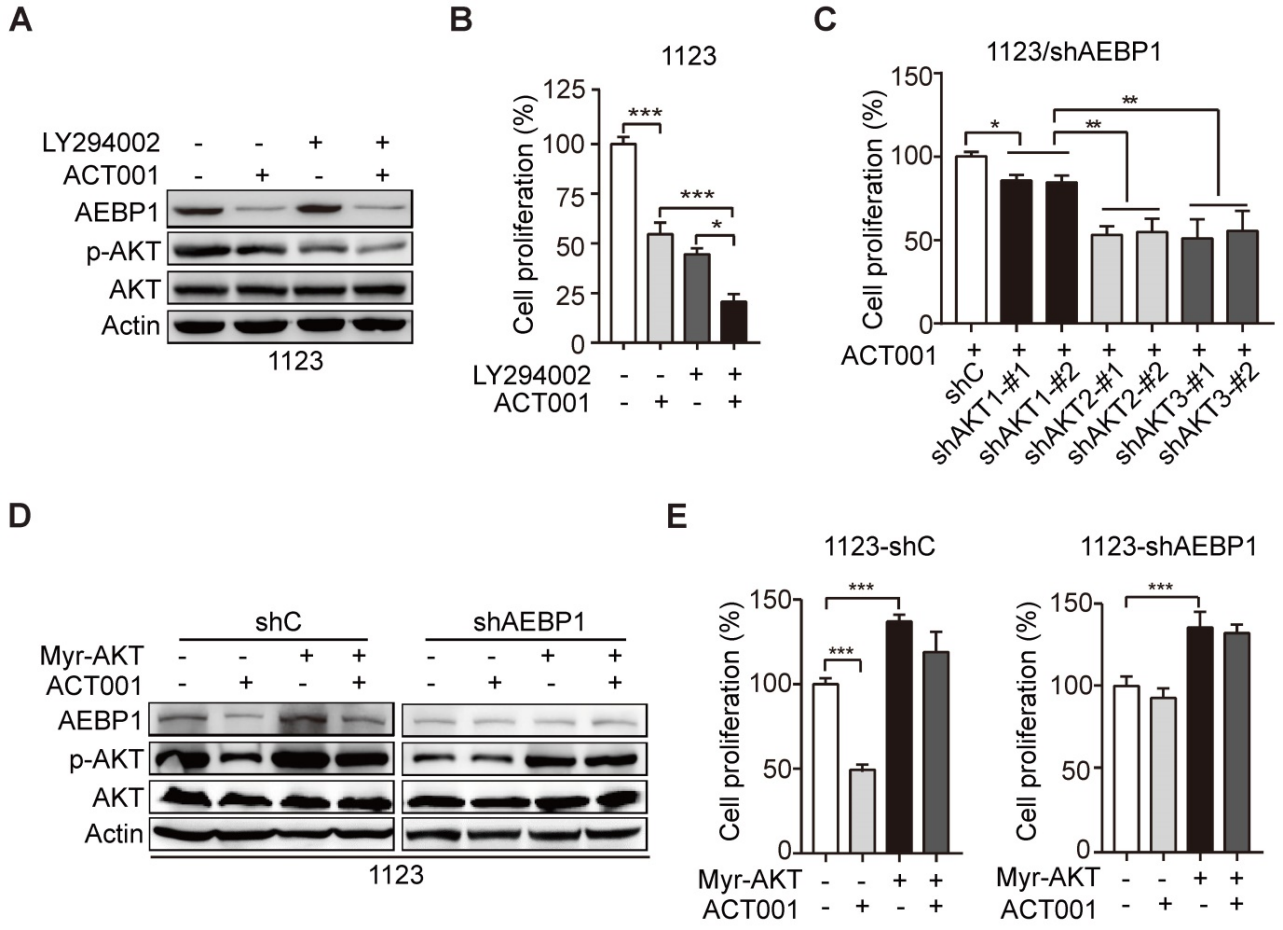
** ACT001 inhibits GSC proliferation through AEBP1/PI3K/AKT signaling. (A)** Effect of PI3K inhibitor LY294002 (10 μM) treatment on ACT001 response in GSCs. GSC 1123 cells were treated with vehicle control (0.01% DMSO), ACT001 (10 μM), or/and LY294002 for 24 h. **(B)** Quantification of cell growth. **(C)** Effects of KD of *AKT1/2/3* on *AEBP1* KD-decreased GSC 1123 cell proliferation. **(D)** Effects of overexpression of a constitutively active AKT mutant (Myr-AKT) on ACT001 response in GSCs. **(E)** Quantification of cell proliferation. Data represent the mean ± SEM of replicates from three independent experiments. *p < 0.05, **p < 0.01, ***p < 0.001, by two-tailed *t*-test.

To further investigate whether ACT001 inhibits GSC proliferation through AEBP1-activated AKT, we treated GSC 1123 cells with PI3K inhibitor LY294002 with or without ACT001. As shown in Figures 4A and 4B, compared to the vehicle control, LY294002 treatment inhibited p-AKT and cell proliferation but not AEBP1 expression. ACT001 treatment in combination with LY294002 further decreased p-AKT and cell proliferation compared with the single treatment of ACT001 or LY294002 (Figures 4A and 4B).

There are three AKT/PKB isoforms, AKT1 (PKBα), AKT2 (PKBβ), and AKT3 (PKBγ) in mammalian genomes 27. To assess which AKT isoform is promoted by AEBP1 in GSCs, we separately transfected the shRNAs of AKT1, AKT2, or AKT3 into GSC 1123/shAEBP1 cells. As shown in Figure 4C, compared to the control, KD of *AKT1*, *AKT2*, or *AKT3* decreased cell proliferation. However, compared to *AKT1* KD, depletion of *AKT2* or *AKT3* significantly decreased cell proliferation in GSC 1123/shAEBP1 cells. This result indicates that *AEBP1* KD partially decreases AKT1, but not AKT2/3 activity and thereby *AKT1* KD weakly inhibits cell proliferation compared to *AKT2/3* KD in *AEBP1* KD cells.

Next, we determined the effects of AKT activation on AEBP1-mediated ACT001 response. As shown in Figures 4D and 4E, compared with the vector control, ectopic expression of a constitutively activated (CA) AKT (Myr-AKT) mutant did affect AEBP1 expression decreased by ACT001 treatment or/and *AEBP1* KD in GSC 1123/shC or 1123/shAEBP1 cells. However, overexpression of the CA AKT mutant rescued ACT001 treatment or/and *AEBP1* KD-decreased p-AKT (Figure 4D) and cell proliferation (Figure 4E). These data indicate that ACT001 inhibits GSC proliferation through AEBP1-activated PI3K/AKT signaling.

### ACT001 inhibits TGF-β-induced AEBP1 expression, GSC proliferation, and glioma sphere formation

**Figure 5:**
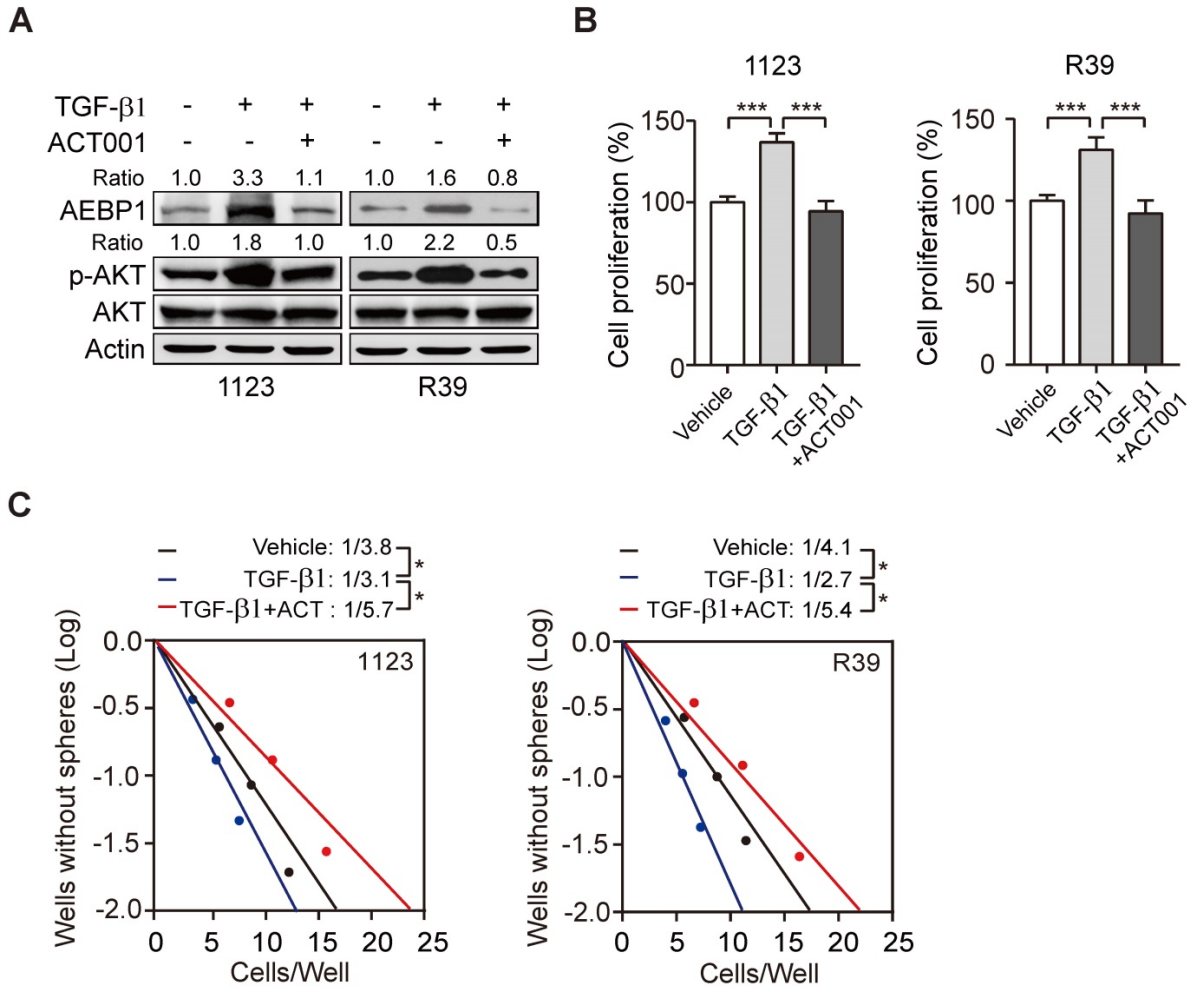
** ACT001 inhibits TGF-β-induced AEBP1 expression, GSC proliferation and glioma sphere formation. (A)** ACT001 inhibits TGF-β-induced AEBP1 expression in GSC 1123 and R39 cells. Cells were treated with vehicle control (PBS), ACT001 (10 μM), or/and TGF-β (20 μg/ml) for 24 h before immunoblotting using the indicated antibodies. **(B)** Quantification of cell proliferation. **(C)** Effects of TGF-β with or without ACT001 in limiting dilution glioma sphere-forming analysis. Data represent the mean ± SEM of replicates from three independent experiments. *p < 0.05, ***p < 0.001, by two-tailed *t*-test.

Transforming growth factor-β (TGF-β) plays important functions in regulating GBM progression and self-renewal 13, 16, 28, high TGF-β activity confers poor prognosis in glioma patients 29, and the oncogenic MSH6-CXCR4-TGF-β1 feedback loop is a novel therapeutic target for GBM 30. Recently we also demonstrated that TGF-β-activated lncRNA LINC00115 as a critical regulator of GSC self-renewal and tumorigenicity 31. Moreover, AEBP1 was shown to be upregulated by TGF-β 32. Thus, we assessed whether ACT001 treatment would impair TGF-β-induced AEBP1 expression and GSC self-renewal. Compared with the control, TGF-β treatment in GSC 1123 and R39 cells induced AEBP1 expression, activated p-AKT (Figure 5A), and promoted cell proliferation (Figure 5B) and glioma sphere formation (Figure 5C). ACT001 treatment significantly attenuated TGF-β-induced AEBP1 expression, p-AKT (Figure 5A), cell proliferation (Figure 5B), and glioma sphere formation (Figure 5C). These data suggest that ACT001 inhibits TGF-β-induced AEBP1 expression, GSC proliferation, and glioma sphere formation.

### ACT001 inhibits GSC tumorigenicity in combination with SHP-2 inhibitor SHP099

**Figure 6:**
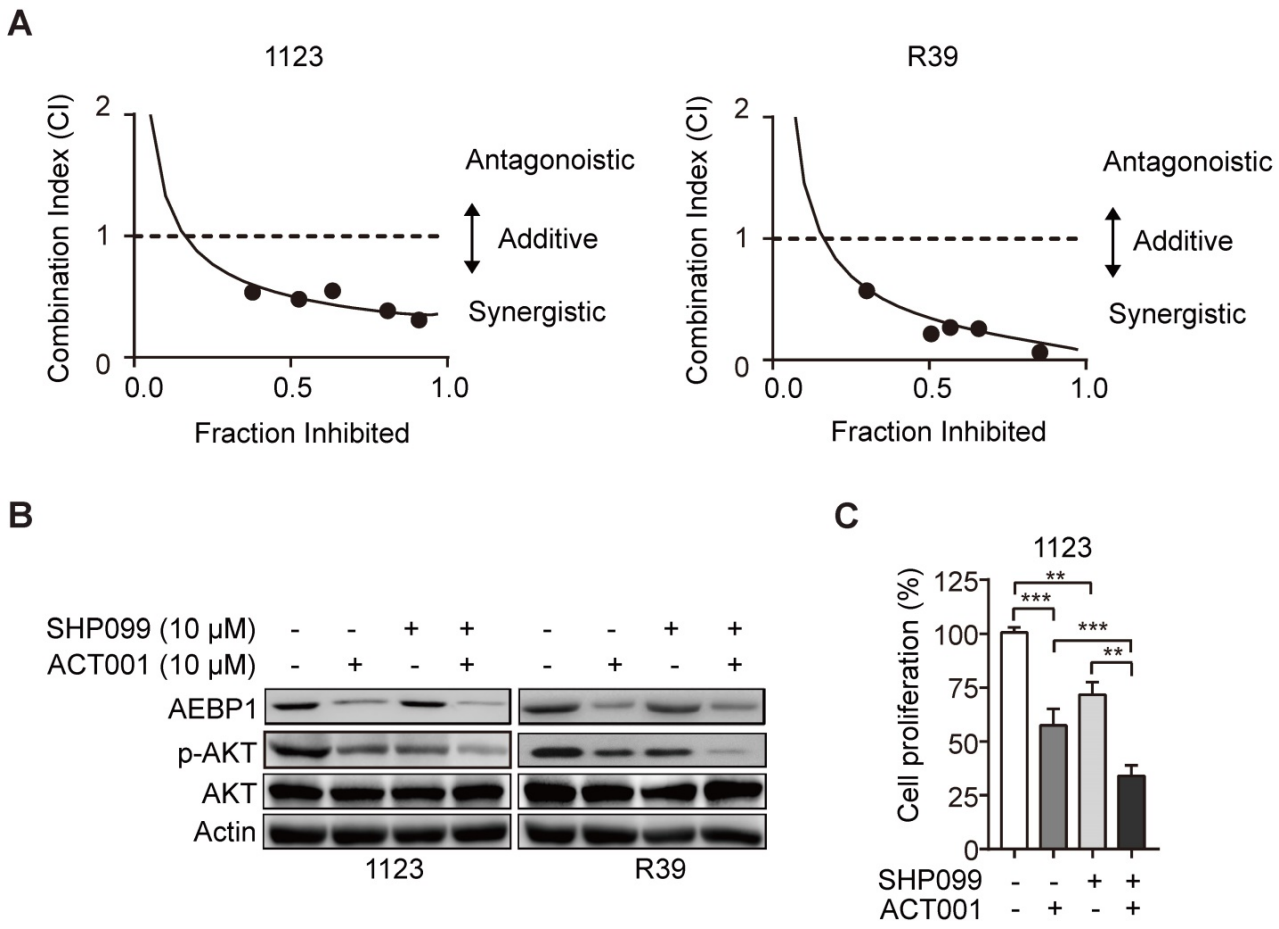
** ACT001 inhibits GSC tumorigenicity in combination with SHP-2 inhibitor SHP099. (A)** Combination index (CI) plots. Fraction affected refers to the proportion of cells with inhibition of viability. Dose response curves of ACT001 combined with SHP009 were determined for each cell line. CI values were calculated using the median-effect equation described by the Chou-Talalay method. CI < 1 indicates synergism, CI = 1 indicates an additive effect, and CI > 1 indicates antagonism. **(B)** ACT001 and SHP099 inhibits AEBP1-AKT expression in GSC 1123 and R39 cells. Cells were treated with vehicle control (PBS), ACT001 (10 μM), or/and SHP099 (10 μM) for 24 h before immunoblotting using the indicated antibodies. **(C)** Quantification of cell growth. Data represent the mean ± SEM of replicates from three independent experiments. **p < 0.01, ***p < 0.001, by two-tailed *t*-test.

SHP099 is a highly potent and orally available inhibitor of SHP-2 and inhibits multiple cancer progression 33-35. Our recent study demonstrated that SHP099 exhibits antitumor activity either as a single agent or in combination with TMZ and provides significant survival benefits for GBM tumor xenograft-bearing animals 20. We first test if the superior effect of ACT001 plus SHP099 by the synergistic effect of the two compounds addition. We used the Chou-Talalay method for drug combination studies^23^ and determined the dose-response curves of ACT001 and SHP099 individually and in combination at a constant potency ratio. For each of the GSC cell lines, we observed the single-agent activity of ACT001 and SHP099 and a remarkably potent synergistic inhibition of cell viability after combined treatment. The calculated combination index (CI) values for GSC 1123 and R39 cells at doses effective in reducing cell viability by 90% were 0.48, 0.21, respectively (Figure 6A). The CI value of ACT001 in combination with SHP099 was less than 1 (< 1), indicating that ACT001 has a synergistic effect with SHP099. We further assessed the activities of AEBP1/AKT axis in response to ACT001 and SHP099 alone or in combination in GSC 1123 and R39 cells. As shown in Figures 6B and 6C, compared to the vehicle control, SHP099 decreased p-AKT and cell proliferation but not AEBP1 expression. Combined treatment with ACT001 and SHP099 significantly decreased p-AKT and cell proliferation compared to single ACT001 or SHP099 treatment (Figure 6A-6C), suggesting that ACT001 in combination with SHP099 decreases GSC proliferation *in vitro*.

**Figure 7:**
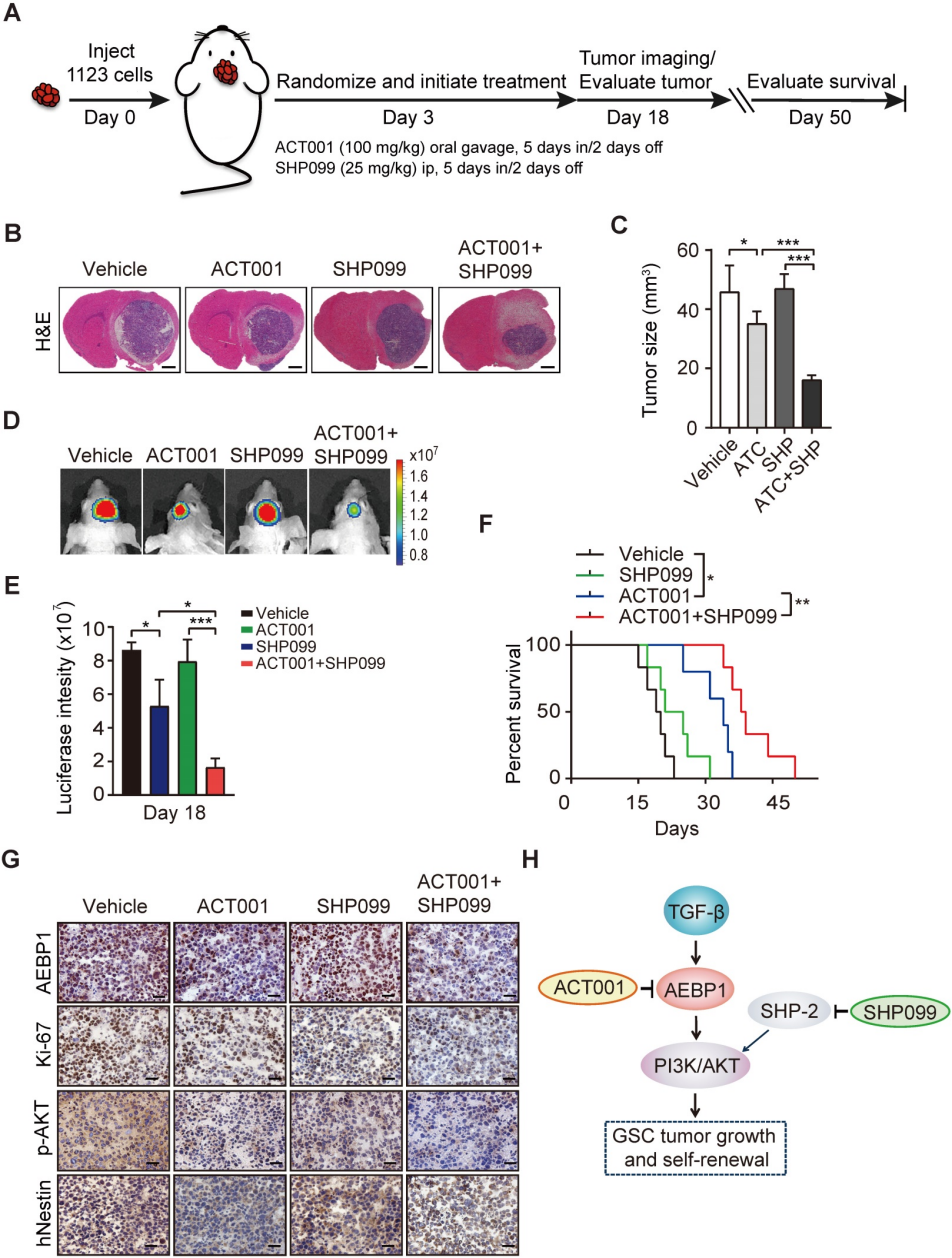
** ACT001 inhibits GSC tumorigenicity in combination with SHP-2 inhibitor SHP099. (A)** Treatment scheme for the evaluation of *in vivo* efficacy of ACT001, SHP099 alone or in combination in GSC 1123 tumor xenografts. **(B)** Representative images on Day 18 after implantation. Scale bars, 1 mm. **(C)** Quantitation of tumor sizes. **(D)** Representative bioluminescence images (BLI) of GSC 1123 xenografts on Day 18. GSC 1123 cells with stable expression of luciferase were used. **(E)** Quantitation of BLI. **(F)** Kaplan-Meier survival analysis of mice with GSC 1123 tumor xenografts (n = 5 or 6). Median survival (in days): Vehicle control, 19.5; SHP099, 23; ACT001, 34; the combination, 38.5. **(G)** Immunohistochemical (IHC) analysis of AEBP1, proliferation (Ki-67), p-AKT, and hNestin in GSC 1123 xenograft tumors. Scale bars, 20 μm. hNestin, an anti- human Nestin antibody.** (H)** A working model of ACT001-targeted TGF-β/AEBP1/AKT signaling pathway in GSC tumor growth and self-renewal. Data represent the mean ± SEM of replicates from three independent experiments. *p < 0.05, **p < 0.01, **p < 0.001, by two-tailed Student's *t*-test or log rank analysis.

Next, to examine the *in vivo* antitumor effect of single ACT001 treatment or in combination with SHP099, we employed an orthotopic xenograft model in immunodeficient mice. Oral gavage of ACT001 (100 mg/kg) with or without intraperitoneal injection of SHP099 (25 mg/kg) was initiated after randomization at Day 3 after GSC 1123 implantation (Figure 7A). Compared to the vehicle control, ACT001 treatment markedly decreased tumor growth (Figures 7B-7E) and extended the survival GBM-bearing animals (Figure 7F). Additionally, IHC analysis showed that ACT001 significantly reduced p-AKT and the expression of AEBP1, cell proliferation marker Ki-67, and GSC marker hNestin (human Nestin) in GSC 1123 xenograft tumors (Figure 7G). ACT001 in combination with SHP099 further reduced tumor burden (Figures 7B-7E), p-AKT, the expression of AEBP1, Ki-67, hNestin (Figure 7G), and extended survival to a greater extent than ACT001 treatment alone (Figure 7F), suggesting that the combination of ACT001 and SHP099 synergizes to extend the survival in our GSC xenograft model (Figure 7H).

## Discussion

Despite ACT001 had exhibited good potential for GBM treatment in clinical trials 12, the mechanism by which ACT001 inhibits tumor growth is still unclear. In this study, we conducted an extensive preclinical *in vitro* and *in vivo* analysis of ACT001 inhibition of multiple GSC cells and one GSC xenograft model. GSCs were more responsive to ACT001 compared with NHA *in vitro*. ACT001 inhibited GSC proliferation and glioma sphere formation through targeting AEBP1/PI3K/AKT signaling and prolonged the survival of animals as a single agent or in combination with SHP099 in an orthotopic GSC xenograft.

ACT001 have shown to be a potential inhibitor for breast cancer 36, Parkinson's disease 37, leukemia 11, 12, and glioma 10, 14, 37. Moreover, ACT001 was demonstrated to selectively inhibit acute myelogenous leukemia stem and progenitor cells 11, 12. Here, our data showed that GSCs are more responsive to ACT001 compared with NHA, further suggesting ACT001's potential in clinical trials.

Biological analyses have established that ACT001 inhibits various tumor growth. However, the mechanism by which ACT001 inhibits cell proliferation remains unclear. ACT001 functioned as a PAI-1 inhibitor and exerted synergistic effects in combination with cisplatin through inhibiting PI3K/AKT pathway in glioma 14. ACT001 also impaired NF-κB activation in breast cancer and glioma cells 36, 37. In this study, we identified AEBP1 as a new target of ACT001. ACT001 treatment decreased AEBP1 expression and AEBP1-activated PI3K/AKT signaling in GSCs. Knockdown of AEBP1 attenuated GSC response to ACT001. PI3K inhibitor treatment sensitized GSCs to ACT001, whereas overexpression of a constitutively activated AKT mutant reduced GSC response to ACT001. ACT001 treatment also blocked TGF-β-induced AEBP1/PI3K/AKT signaling.

Taken together, this study identifies AEBP1 as a new target of ACT001 and a putative prognostic factor in glioma. The newly elucidated roles of AEBP1 in GSC proliferation and glioma sphere formation provide a strong rationale for targeting this molecule in clinical treatment of human gliomas. ACT001 as a single agent or in combination with SHP-2 inhibitor SHP099 provides significant survival benefits to GBM tumor xenograft-bearing animals, which also provide a strong rationale for validation of combination of ACT001 and SHP099 as a therapeutic approach against GBM.

## Supplementary Material

Supplementary figures.
