# Supplementary material for: Targeting of glioma stem-like cells with a parthenolide derivative ACT001 through inhibition of AEBP1/PI3K/AKT signaling
Source: Theranostics. 2021 Jan 1;11(2):555–66. doi: 10.7150/thno.49250 (PMC7738851; doi:10.7150/thno.49250)
Supplement: Supplementary file 1 — Supplementary figures. [file thnov11p0555s1.pdf]

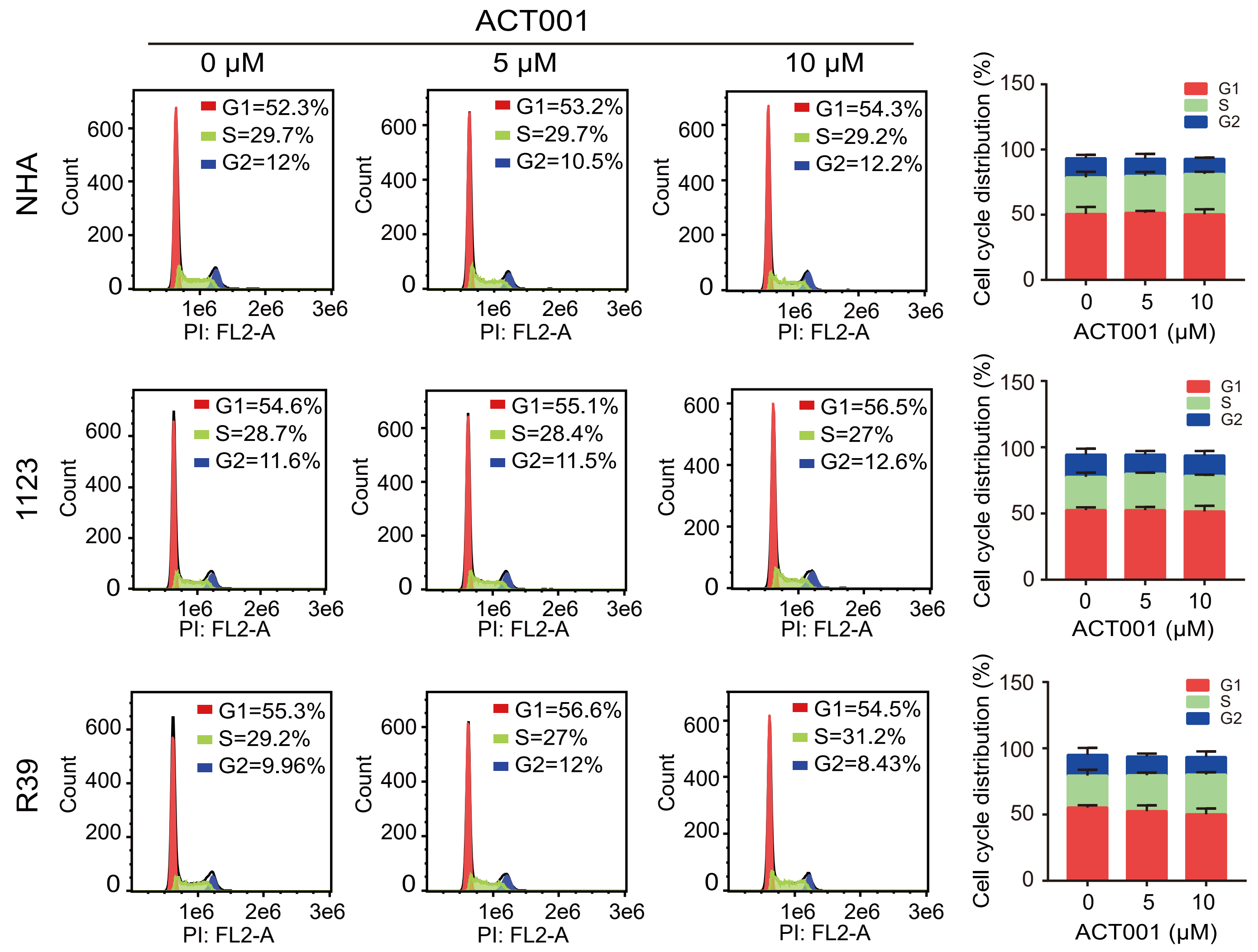

Figure S1 Effects of ACT001 on cell cycle in NHA, GSC 1123, or R39 cells

NHA, GSC 1123 or R39 cells were treated with 0, 5, or 10 μM ACT001 for 24 h. Quantitative analysis of the cell cycle distributions were performed. Data represent the mean ± SEM of replicates from 3 independent experiments, by two-way ANOVA.

Supplementary Figure 2 Original WB

Figure S2D

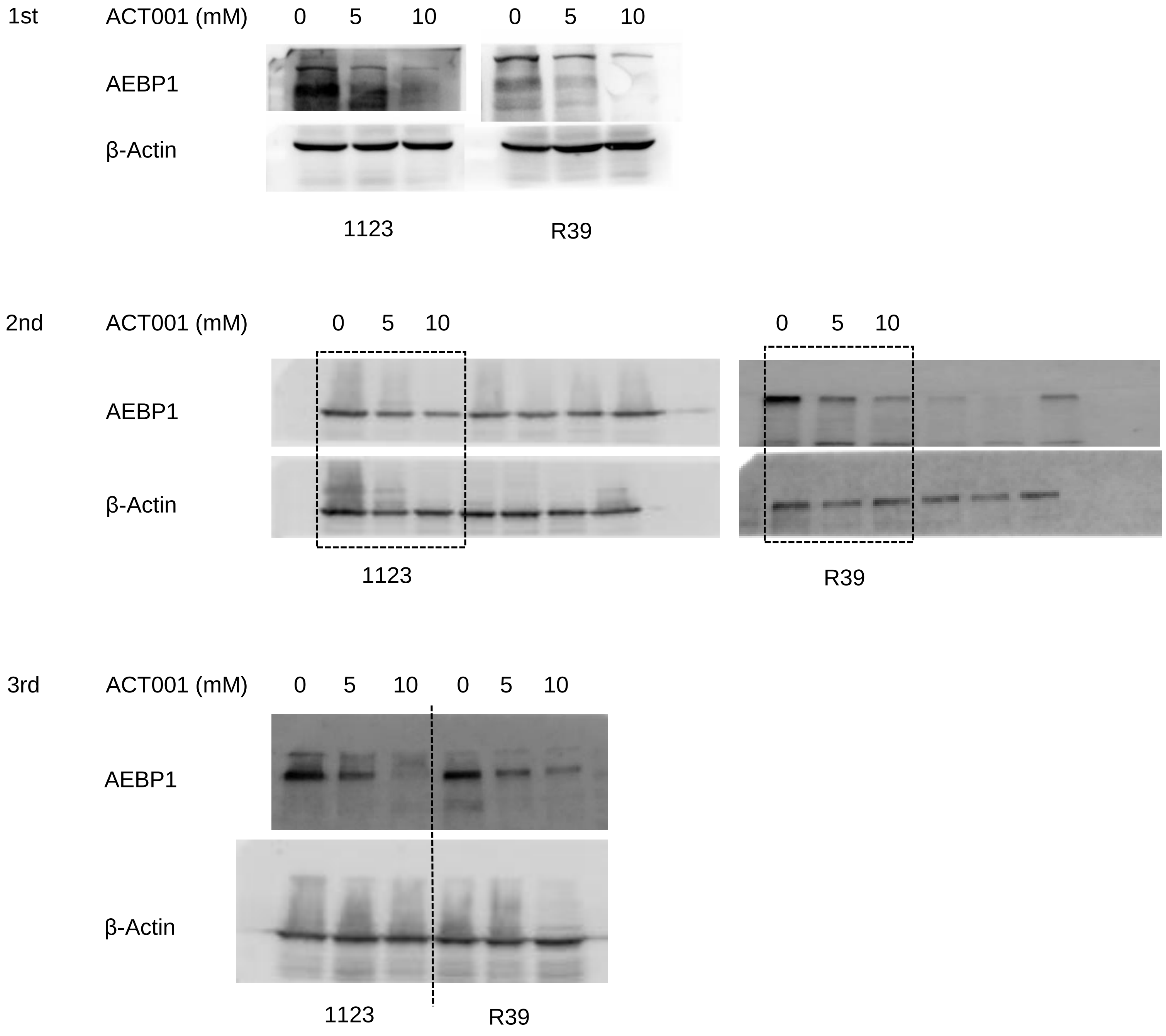

Figure S3A

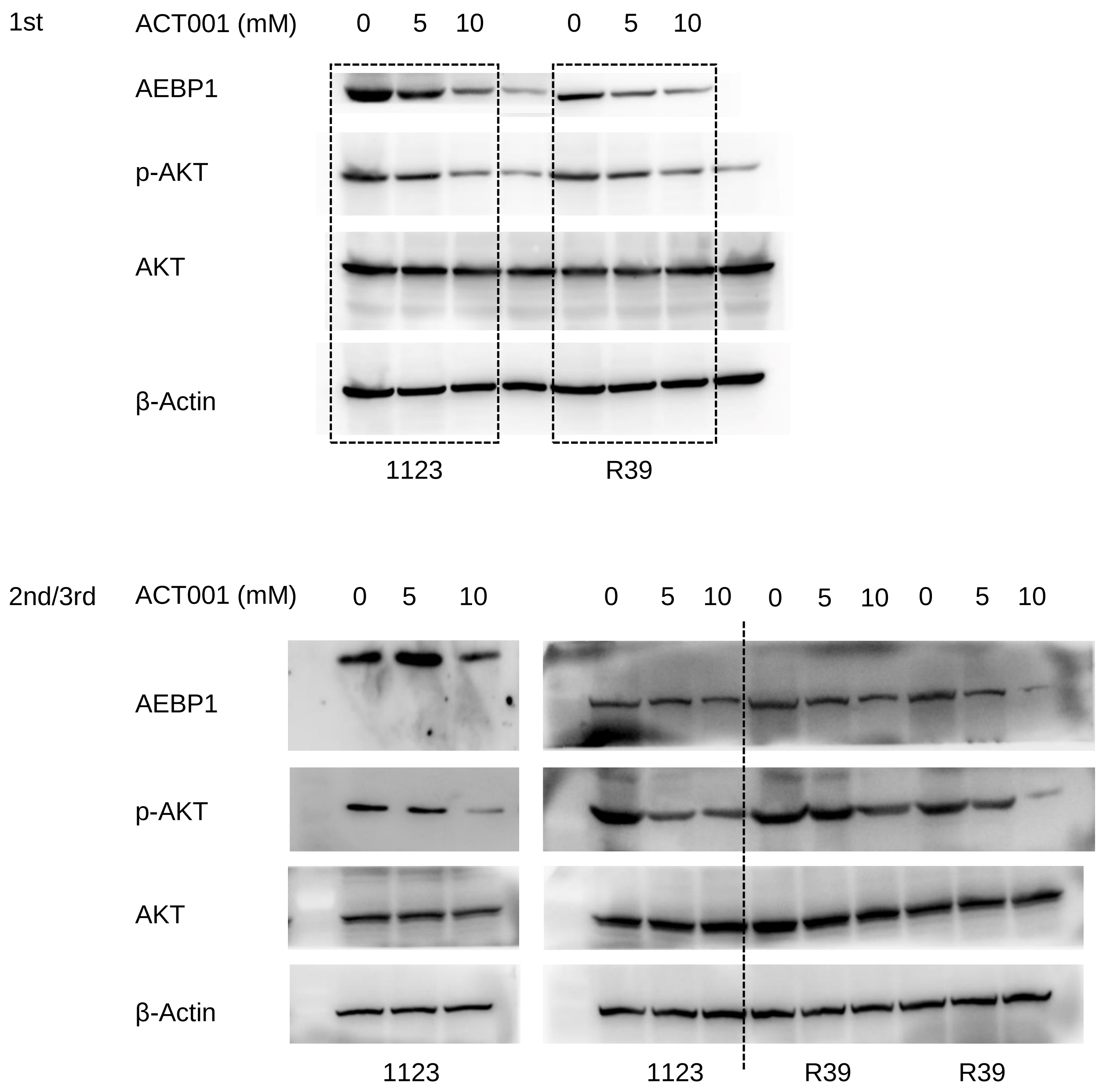
1st
ACT001 (mM)
0 5 10 0 5 10
AEBP1
p-AKT
AKT
β-Actin
1123
R39
2nd/3rd
ACT001 (mM)
0 5 10
0 5 10 0 5 10 0 5 10
AEBP1
p-AKT
AKT
β-Actin
1123
1123
R39
R39

Figure S5A

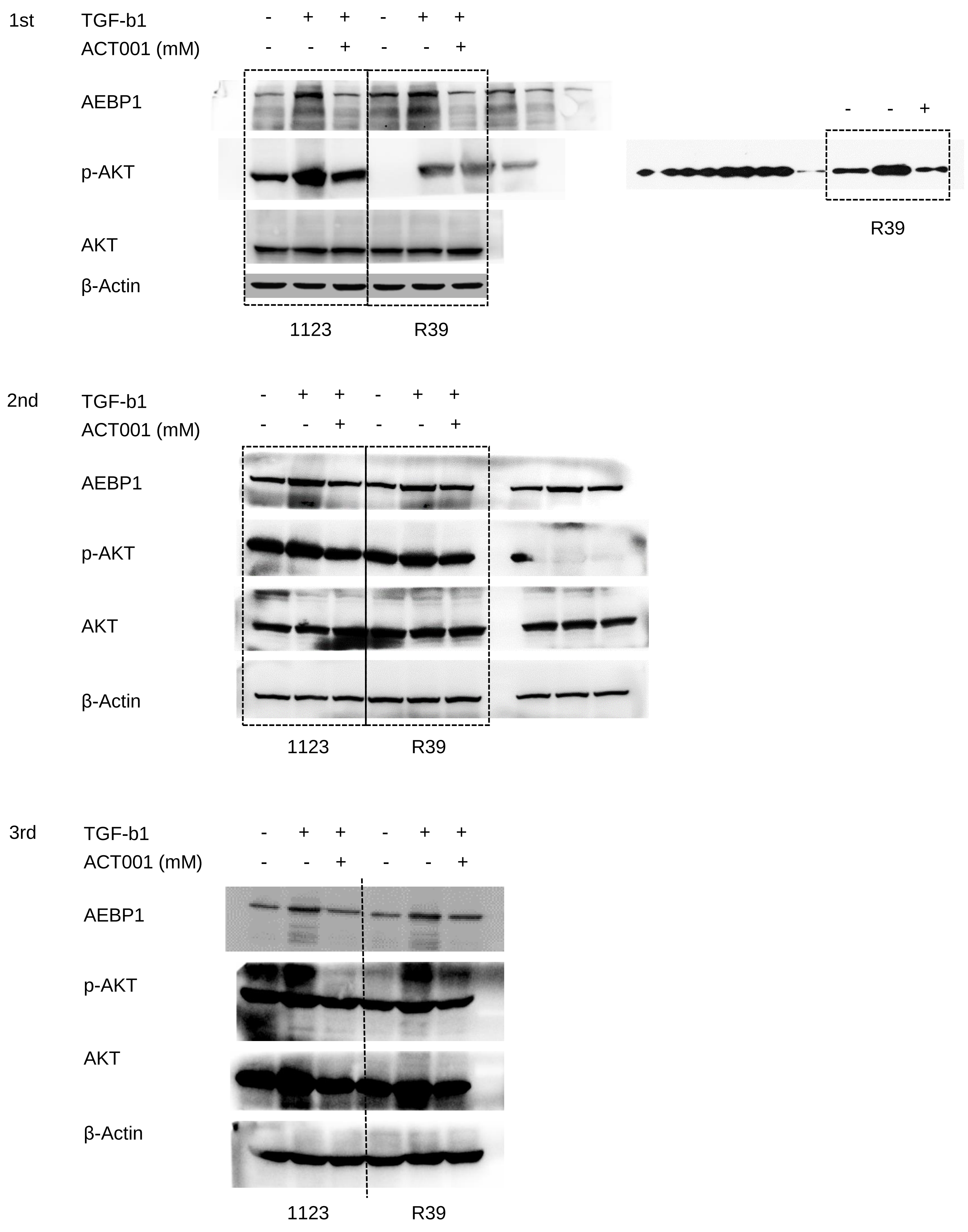
1st
TGF-b1
ACT001 (mM)
AEBP1
p-AKT
AKT
β-Actin
1123
R39
R39
2nd
TGF-b1
ACT001 (mM)
AEBP1
p-AKT
AKT
β-Actin
1123
R39
3rd
TGF-b1
ACT001 (mM)
AEBP1
p-AKT
AKT
β-Actin
1123
R39
